# A similar correction mechanism in slow and fluent readers after suboptimal landing positions

**DOI:** 10.3389/fnhum.2014.00355

**Published:** 2014-06-03

**Authors:** Benjamin Gagl, Stefan Hawelka, Florian Hutzler

**Affiliations:** Department of Psychology, Centre for Neurocognitive Research, University of SalzburgSalzburg, Austria

**Keywords:** eye movements, reading, landing position, slow readers, corrective re-fixations

## Abstract

The present eye movements study investigated the optimal viewing position (OVP) and inverted-optimal viewing position (I-OVP) effects in slow readers. The basis of these effects is a phenomenon called corrective re-fixations, which describes a short saccade from a suboptimal landing position (word beginning or end) to the center of the word. The present study found corrective re-fixations in slow readers, which was evident from the I-OVP effects in first fixation durations, the OVP effect in number of fixations and the OVP effect in re-fixation probability. The main result is that slow readers, despite being characterized by a fragmented eye movement pattern during reading, nevertheless share an intact mechanism for performing corrective re-fixations. This correction mechanism is not linked to linguistic processing, but to visual and oculomotor processes, which suggests the integrity of oculomotor and visual processes in slow readers.

## INTRODUCTION

**FIGURE 1:**
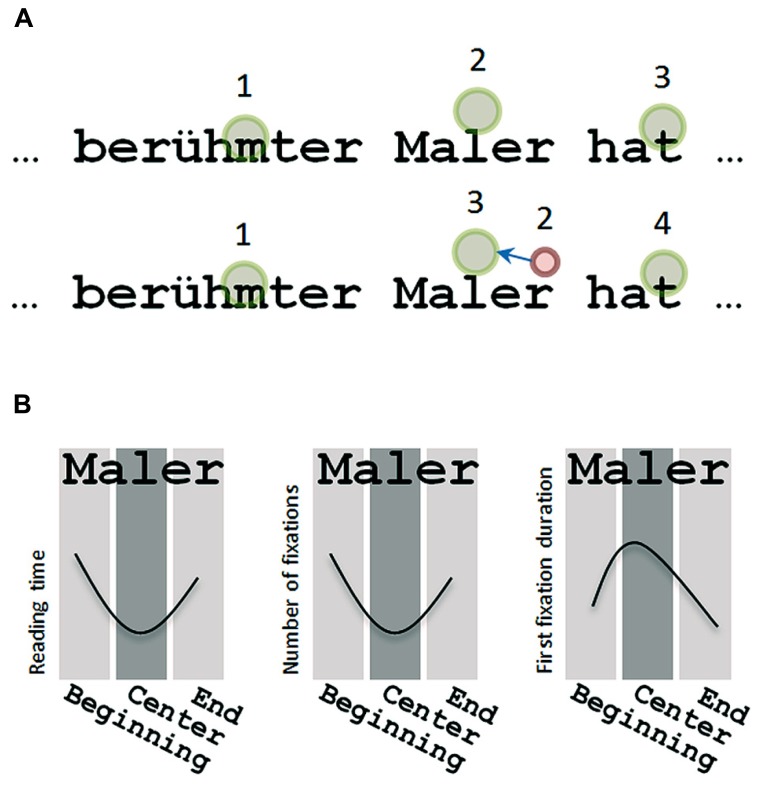
**(A)** Schematic illustration of eye movements during reading. Standard reading fixations are presented as green circles and the numerals indicate the sequential order. In the lower line a corrective re-fixation occurs after suboptimal landing at the end of the word *Maler.* The initial fixation on *Maler*, presented in a smaller red circle (indicating a shorter fixation duration), is followed by a saccade (blue arrow) to set up a fixation near the word center (Hutzler et al., 2008). **(B)** The optimal viewing position effects (OVP effect) for reading times and the number of fixations for landing positions at the beginning, the center and the end of the word *Maler*. The right panel depicts the inverted optimal viewing position effect (I-OVP effect) for first fixation durations. The OVP effect shows longer reading times and a higher number of fixations after landing at the word beginning or the word end. In contrast, the I-OVP effect on first fixation durations shows the shortest fixation durations at word beginnings or ends and the longest durations at the word center. Note that, after landing at the word center of a short to medium length word (e.g., smaller than eight letters) only one fixation is typically required for word recognition (e.g., Vitu et al., 2001).

The initial landing position of the eyes on a word affects the speed of word recognition (e.g., O’Regan and Jacobs, 1992; Hutzler et al., 2008). Typically, a fixation position slightly left to the word center, which is termed as the optimal viewing position (OVP), allows fast word recognition. In contrast, landing on the initial or the final letters of a word increases word processing times (see **Figure 1**). This is a classical finding from visual word recognition research, which could be replicated in different languages (Finnish: Hyönä and Bertram, 2011; German: Hutzler et al., 2008; French: O’Regan and Jacobs, 1992; Vitu et al., 2007) and different age groups (e.g., Aghababian and Nazir, 2000). Eye movement evidence suggests that increased reading times at suboptimal landing positions are the result of a correction mechanism that precedes visual word recognition to provide high quality visual information to the reading system. **Figure 1A** (bottom) presents a schematic example of a corrective re-fixation. Also, in **Figure 1B**, a schematic description of the OVP effect in reading time is presented and effects on eye movements that are described in the following.

In reading paradigms, for example, silent sentence or passage reading, word processing measures such as the number of fixations and the percentage of re-fixations typically show an OVP effect. The number of fixations and the percentage of re-fixations are lowest after landing at the word center when compared to suboptimal landing positions (**Figure 1B**; e.g., McConkie et al., 1989; Vitu et al., 2001; Nuthmann et al., 2005). Similar OVP effects were found for gaze duration. In addition, the study of Vitu et al. (2001) described the influence of landing position on the first fixation duration. The initial fixation duration showed an inverted-optimal viewing position effect (I-OVP) with the longest fixation durations at the center of the word and shorter fixations at suboptimal landing positions, that is, the word beginning or end (see **Figure 1B**). This finding was replicated in different languages (e.g., Vitu et al., 2001; Nuthmann et al., 2005; Hyönä and Bertram, 2011), age groups (Vitu et al., 2001; Huestegge et al., 2009; Joseph et al., 2009), and successfully modeled for adults (Reichle et al., 2003; Engbert et al., 2005) and children (Reichle et al., 2013).

At first sight, the I-OVP effect was counterintuitive since the initial fixation duration showed the opposite pattern compared to reading times (**Figure 1B**). To understand the I-OVP effect, the complementary OVP effects on the number of fixations and the probability of re-fixating a word, have to be taken into account. The first line of **Figure 1A** shows a standard case where a word is fixated at a preferred central location (Rayner, 1979) and recognized by a single fixation. In this case the fixation durations are the longest and influenced by linguistic word characteristics (e.g., Vitu et al., 2001). In the second line of **Figure 1A** the word *Maler* is initially fixated at the word end. After such a suboptimal fixation position the duration is typically short and accompanied by a re-fixation at the word center. The initial short fixations are not influenced by word characteristics such as word frequency (Vitu et al., 2001; Vergilino-Perez et al., 2004) or the lexicality of the letter string (i.e., word vs. pseudoword; Hutzler et al., 2008). To summarize, when readers land optimally, the initial fixation durations are longer, influenced by the linguistic properties of the word and less likely followed by an additional fixation. In case of landing at the beginning or the end of a word, initial fixation durations are shorter, not influenced by linguistic word characteristics and highly likely followed by a re-fixation.

The mechanism underlying the I-OVP–OVP effect combination is a correction process that initiates a saccade from unfavorable landing positions to the center of a word (see **Figure 1A**). This initial information processing and saccade programming is independent from linguistic processing (Hutzler et al., 2008). In other words, after landing at an unfavorable landing position the brain recognizes that the position is off target (i.e., word center) and corrects the position by means of a fast eye movement towards the preferred location near the word center. In a study by Hutzler et al. (2008), this mechanism was labeled as corrective re-fixations and might allow the investigation of non-linguistic processing, such as visual and oculomotor processing of slow readers.

Landing position effects on slow readers (e.g., dyslexic readers) were seldom reported and, to our knowledge, there is no existing report of an I-OVP effect in fixation durations and OVP effects in the number of fixations and the re-fixation probability. Two eye movement studies (MacKeben et al., 2004; Hawelka et al., 2010) reported landing position data of dyslexic readers. These readers tend to target the word beginning more often than fluent readers. In addition, the relation between initial landing position and word length was investigated. Here the classical finding is that a fluent reader tries to initially fixate a position near the word center (Rayner, 1979). This means that for a short word of four letters the preferred viewing location is near the second letter but on an eight-letter word the preferred viewing location would be around the fourth letter. In the dyslexic readers of Hawelka et al. (2010) the influence of word length on the initial landing position was smaller than in the fluent readers. They landed more towards the word beginning (at the second letter of words with four to seven letters) than the fluent readers (who landed on the third letter; see also MacKeben et al., 2004). To our knowledge, no further investigation of, for example, a reading time measure in relation to landing position is present in the literature.

The participants of the Hawelka study were German dyslexic readers. In German, dyslexia is mainly characterized by massively impaired reading speed (e.g., Wimmer, 1993). The speed impairment is reflected in prolonged fixation durations as well as in a higher number of fixations per word in comparison to fluent readers (Hutzler and Wimmer, 2004; Dürrwächter et al., 2010; Hawelka et al., 2010). Furthermore, the slow readers exhibited markedly increased word length and frequency effects on number of fixations. In combination, the strong word length effect (e.g., in gaze durations), the high number of fixations and the initial landing position at the word beginnings of slow readers were interpreted as a serial reading strategy. Serial reading is typically present in beginning readers and reflects letter to sound conversion (e.g., Share, 1995; Ziegler and Goswami, 2005). The converted sounds are then assembled to allow access to phonological representations. After the initial phase of literacy acquisition the length effect typically decreases which is interpreted as reflecting the emergence of whole word recognition (see Rau et al., 2014, for a recent eye movement study).

Dyslexic and slow readers might stick to serial reading. From this perspective, word beginnings are reasonable targets for the initial fixation. The most prominent theory, the phonological deficit hypothesis, suggests that in dyslexic individuals the representation, storage, and retrieval of speech sounds is impaired (e.g., Snowling, 2000). Other hypotheses assume impairments in the process of connecting letters and orthographic information (e.g., an orthographic word unit) to the respective phonological representation (e.g., Wolf and Bowers, 1999; Wimmer and Schurz, 2010). These hypotheses are concerned with cognitive processes that are specific for linguistic processing during reading. Another type of deficit theories is concerned with processes during reading apart from the core linguistic processes. Examples would be deficits in visual processing (e.g., magnocellular vision; Stein and Walsh, 1997) or oculomotor processes (e.g., Pavlidis, 1981; Bucci et al., 2008; but see Kirkby et al., 2011). These processes are non-linguistic processes that accompany the core linguistic processes but are not exclusive to reading.

The main aim of the study is investigating the phenomenon of corrective re-fixations in slow readers. In particular, it is investigated whether or not slow readers also show corrective re-fixations along with OVP and I-OVP effects. If slow readers correct for unfavorable landing positions, then they should show an (1) OVP effect in the re-fixation probability, (2) OVP effect in the number of fixations and (3) an I-OVP effect on first fixation durations. In case the pattern of effects suggests such a correction procedure, it is of interest if the first fixation durations at suboptimal landing positions are (4) comparable between groups and if they are (5) influenced by word frequency or predictability. An absence of a correction mechanism as well as differences to the normal corrective pattern such as longer fixation durations at suboptimal landing positions would indicate an impairment in the non-linguistic visuo-oculomotor components of reading. For landing positions at the word center, slower readers are expected to show increased fixation durations and stronger effects of frequency and predictability in contrast to fluent readers. With regard to the deficit theories, a comparable correction mechanism would support theories which assume deficient linguistic processing. In contrast, differences in corrective re-fixations would support theories suggesting deficits in non-linguistic processes. Final analyses will investigate the OVP and I-OVP effects for each individual reader (see e.g., Ramus et al., 2003). These analyses will inform whether deficits can be generalized for slow readers or whether there is a distinct subgroup of slow readers who exhibit evidence for a visuo-oculomotor deficit.

## MATERIALS AND METHODS

### PARTICIPANTS

We recorded eye movements from 46 slow readers (18 adolescent dyslexic readers were from Hawelka et al., 2010; 16 student dyslexic readers and 12 academic slow readers from previously unpublished datasets) and 99 fluent readers (18 adolescent fluent readers were from Hawelka et al., 2010 and 49 from Gagl et al., 2011; 28 fluent reading students, from previously unpublished datasets). All readers were either adolescents or adults (16–47 years old) and native speakers of German. All slow readers scored below a percentile of 10 on a reading speed test, which is an adaptation of a reading speed test for children (Auer et al., 2004) and our group is currently collecting the norming samples. The preliminary norms of the test are based on a sample of 309 students. In this test, readers are instructed to mark sentences as semantically correct or incorrect and the number of correctly marked sentences within 3 min was used as a measure of reading speed. For example sentences like “*People with pale skin and blond hair have an enhanced risk of sunburn*” or “*A weighing-machine measures the height of a person*” were included. In addition, all adult slow readers had achieved a high level of education and all adolescent dyslexics had a normal to high IQ. For 34 slow readers (i.e., the two dyslexic groups) two non-verbal subtests of German version of the Wechsler Adult intelligence scale (WAIS-R; German Version: Tewes, 1991) were administered. The group scores (standard deviations) were 12.3 (2.7) and 13.3 (2.8) for block design and object assembly, respectively. Note both scores were higher than the norm mean of 10 (3). This is a typical profile for a group of developmental dyslexics (e.g., Snowling, 2000). In addition, the adolescent dyslexic readers of Hawelka et al. stem from two large scale longitudinal studies from Salzburg (e.g., Wimmer et al., 2000) and most of the student dyslexic readers were diagnosed with developmental dyslexia before (10/16). The third group of 12 adult slow readers achieved at least a higher education entrance qualification. They were students or already hold an academic degree and therefore it is highly unlikely that their reading speed deficit is due to an intellectual handicap. To be conservative we refer to the whole group as slow (rather than as dyslexic) readers.

Fluent readers were included, if they exhibited a reading score above percentile 35. As an additional measure for the group selection we calculated a word per minute measure (wpm) from the eye movement sentence reading task. Similar to Rayner et al. (2010) we set a *wpm* criterion, which was 200 wpm. Eight slow readers (seven from the student dyslexics and one slow reading academic) and 17 fluent readers performed above and below the criterion, respectively. Thus they might be inadequately assigned to their reading group. To be conservative, we discarded these participants from further analysis. As a result 38 slow and 82 fluent readers (*M* = 139 wpm; SD = 38 and 277 wpm; SD = 55, respectively) were included in the final analysis.

### MATERIALS

Participants read the 144 sentences with various grammatical structures of the Potsdam sentence corpus (PSC; Kliegl et al., 2004), which were presented in a mono-spaced, bold Courier New font (14 pt; 0.3° character width). Eye movements were analyzed on all words with four to seven letters (*n* = 495 words; *M* = 5.5 letters; SD = 1.1; four-letter words: *n* = 121; five-letter: 135; six-letter: 125; seven-letter: 114). We note that more than one word per sentence were included in the analysis. For example, in the sentence “*Der **Gehilfe** des Gärtners sät **Kresse** und Radieschen*.” the words in bold letters met the criteria. However, the initial word of a sentence was not considered for analyses. Predictability measures were provided by the Potsdam group and word frequency values were obtained from the SUBTLEX frequency norms (Brysbaert et al., 2011). The mean log SUBTLEX frequency of the target words was 3.36 (SD = 1.17) per million and the mean predictability was 0.19 (SD = 0.29) in the whole set of the corpus sentences. The dyslexic student readers and their control group (*n* = 9 and *n* = 22, respectively) read a reduced set of the Potsdam sentence corpus (*n* = 36 sentences). In this short version, 157 words of four to seven letters were analyzed (*M* = 5.5 letters; SD = 1.1; *n* = 32, 48, 41, and 36 for the levels of words lengths, respectively). These words had a very similar frequency and predictability as those from the entire corpus (*M* = 3.36; SD = 1.10; *M* = 0.19; SD = 0.28, respectively).

### APPARATUS AND PROCEDURE

The eye movement recordings and the sentence reading paradigm are described in detail in Hawelka et al. (2010) and in Gagl et al. (2011). In short, an Eyelink 1000 tower mount system (SR-Research, Ontario, Canada) was used to record the eye movements of the right eye. The participant’s heads were placed in a head and a chin rest in front of a 21’ cathode ray tube monitor (Belinea, Germany, 1024 × 768 screen resolution; 120 Hz refresh rate). The sentence reading task was preceded by a monocular calibration procedure and 10 practice sentences. The participants were instructed to read the sentences silently. The presentation of the sentences was triggered by a fixation at a fixation point at the left side of the screen (vertically centered). After the detection of a fixation at the fixation point, a sentence was presented in such a way that the fixation landed at the OVP of the first word of the sentence. To terminate the sentence presentation a cross at the bottom right corner of the screen had to be fixated whereupon a new trial was initiated. After about a quarter of the sentences a comprehension question was orally presented. These questions could mostly be answered with a single word. To ensure high eye tracking quality and a low number of recalibrations (in both groups on average four times) the chin rest was placed in such a way that the utterance was not hampered. Both groups had no problems comprehending the sentences, which was reflected in their nearly perfect performance on the comprehension questions (>98% in both groups).

### DATA TREATMENT AND ANALYSIS

First fixation durations, the re-fixation probability and the number of fixations were analyzed during the first pass reading of all 4–7 letter words in relation to the initial landing position. All fixation durations shorter than 80 ms and longer than 800 ms were removed from analysis (<2% of the fixation durations in each group). In sum 14,994 and 27,865 fixations were analyzed for slow and fluent readers, respectively, of which 4,597 and 6,043 landed at the beginnings of words and 1,028 and 5,054 landed at the ends of words. The re-fixation probability is estimated by setting the probability to one in case a word was re-fixated (i.e., more than one fixation) and to zero in single fixation cases. The re-fixation probability of, for example, a specific word would then be the mean of the probability values from each participant for this word excluding cases in which the word was skipped.

Data analysis was performed with linear mixed effect models (LMM) with the *lme4* package (Bates et al., 2007) in *R*. The LMM analysis is suitable for investigating sentence corpus data since LMMs deal well with missing data and they allow treating items and participants as random effects in a single analysis. For fixation durations, we analyzed both untransformed and log-transformed durations. The result of such a transformation usually lead to a better fit between observed and predicted data (i.e., to smaller residuals). However, we did not observe differences in the pattern of effects for the two analyses. Thus, we report the coefficients and statistics of the untransformed data from which one can more readily perceive the effect sizes. For the analyses of re-fixations and number of fixations the binomial and the Poisson distributions were used for modeling the LMMs, respectively.

To capture the parabolic shape of the I-OVP and the OVP effects we centered the first fixation position measure and added in the LMM the second order polynomial term of the centered position (i.e., the squared centered first fixation position). To illustrate, a centered initial landing position of zero relates to either the middle letter of a word or to the space between the two middle letters (in words with an even number of letters). Thus, zero would relate to the third letter in five-letter words or to the position in between the second and the third letter in four letter words. A first fixation position at the second and third letter of a five-letter word is one for both instances (the square of -1 and +1, respectively) and a fixation at the first and the last letter is four (the square of -2 and 2). This convention made possible to capture the parabolic shape of the OVP/I-OVP effects by accounting for the decrease or the increase of the eye movement measures with increasing distance of the fixation position from the word center (see also McConkie et al., 1989; Nuthmann et al., 2005).

To summarize, the main LMM analysis contains a first and second order polynomial of centered first fixation position (i.e., the linear and the squared effect of first fixation position) and the factor reading group. For these three fixed effects all possible interactions were modeled. In addition, we added word frequency, predictability and length as fixed effects to account for the effects of these word characteristics. Participants and items were treated as random effects.

## RESULTS

### GLOBAL EYE MOVEMENT CHARACTERISTICS

The global eye movement characteristics of the slow and the fast readers for all words of the sentence corpus are presented in **Table 1**. As evident from the Table, the slow readers exhibited longer first fixation durations, longer gaze durations and a higher number of fixations. The higher number of fixations was due to a lower percentage of word skippings (7%; fast readers: 20%) and a higher percentage of instances in which words receive multiple fixations (52 vs. 24%). Importantly, for the analysis of re-fixation cases, the total number of cases in which words were fixated more than once is similar in both groups (around 7,500 cases). The initial and second landing position was closer to the word beginnings in the slow readers compared to the fast readers.

**Table 1:** Means (standard errors) of global eye movement measures and group comparisons.

	Slow Readers	Fluent Readers	Group comparisons	
			*t*(118)	*p*
First fixation position (letter)	2.32 (0.05)	2.92 (0.03)	9.2	<0.001
First fixation duration (ms)	236 (5)	193 (2)	9.4	<0.001
Second fixation position (letter)	3.20 (0.11)	3.63 (0.07)	3.4	<0.001
Second fixation duration (ms)	217 (7)	178 (2)	9.4	<0.001
Gaze duration (ms)	512 (40)	284 (11)	7.3	<0.001
Number of fixations	2.37 (0.18)	1.27 (0.05)	7.7	<0.001
Word skipping	7%	20%		
Single fixation cases	41%	56%		
Multiple fixation cases	52%	24%		

*Fixation positions are presented in absolute letter position and fixation probabilities in percentages.*

### LANDING POSITION EFFECTS

**FIGURE 2:**
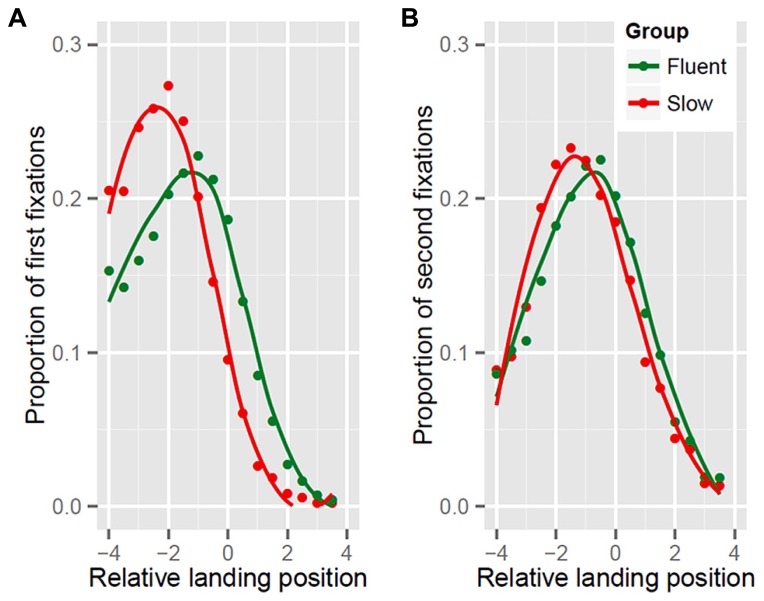
**Landing position distributions of the initial (A) and second fixation position (B) for slow and fluent readers.** The initial landing position is presented in relation to the center of the words.

**Figure 2** presents the landing position distributions of the initial and the second fixation for the four to seven-letter words. Slow readers preferentially targeted the beginning of the words (**Figure 2A**). In contrast, the fluent readers’ initial landing position was, on average, closer to the word center. However, the peak of the distribution was still slightly left of center. Fluent readers did not only target the word beginning less often, they also showed a higher preference for landing positions between the center and the end of a word when contrasted to slow readers. In **Figure 2B**, the distribution of the second fixation position is presented. Here the group differences are more subtle, but still reliable (see **Table 1**). A detailed inspection revealed that the slow readers still tend to fixate slightly more to the left of the center than the fluent readers. The latter fixated more often to the right of the center. Overall however, the second fixation distribution of both groups has its maximum at a position slightly left to the center of the words. Thus, we may assume that the target of the first re-fixation in both groups is near the center of a word.

**FIGURE 3:**
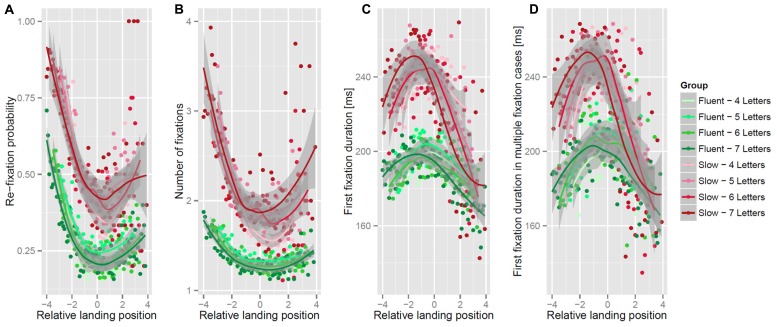
**Re-fixation probability (A) number of fixations (B), first fixation duration (C) and the first fixation duration of multiple fixation cases (D) in relation to centered initial landing position for both groups and all four word length levels.** The lines depicts smoothed means; the gray areas confidence intervals as provided by the ggplot2-package (Wickham and Chang, 2013).

**Figure 3** shows all eye movement measures in relation to centered first fixation position for both groups and all four word length levels. Visual inspection of the I-OVP and OVP effects for each word length indicate that the shape, of the largely overlapping effects, was comparable between length levels. To decrease complexity word length was only added as fixed effect to the LMMs as a control variable without the interactions. The word length effect, on top of the effects of group, the linear and the squared effect of first fixation position, was not reliable for first fixation durations (*t* < 1), re-fixation probability and number of fixations (both *Z*’s < 1.9). **Figures 3A,B** shows the re-fixation probability and the number of fixations in relation to the initial landing position. Increased re-fixation probabilities and number of fixations were found after landing at the word beginning or after landing at word ends. This OVP effect was present for slow and fluent readers. However, the slow readers exhibited more fixations and a higher probability for re-fixations than the fluent readers. The LMM analysis (**Table 2**) confirmed this observation for number of fixations and the re-fixation probability with a reliable effect of group and reliable effects of the linear and squared first fixation position. In addition, both measures showed a reliable interaction of group and linear landing position, which were due to more pronounced linear effects of landing position for the slow than for the fast readers. To be specific, the slow readers, in contrast to the fast readers, exhibited a higher number of fixations and a higher re-fixation probability at word beginnings than at word ends. Critically, no reliable interaction of group and the squared first fixation position was found which indicates that the quadratic effects were similar in both groups.

**Table 2:** Results from the LMM analysis for the percentage of re-fixations, number of fixations, first fixation durations and first fixation durations of multiple fixation cases.

	Percentage of re-fixations			Number of fixations			First fixation duration			First fixation duration of multiple fixations		
	*FE*	*SE*	*z*	*FE*	*SE*	*z*	*FE*	*SE*	*t*	*FE*	*SE*	*t*
Group (G)	**-1.450**	**0.4**	**3.5**	**-0.37**	**0.07**	**5.8**	**-46.3**	**4.4**	**10.5**	**-38.2**	**5.1**	**7.5**
First fixation position (FP)	**-0.271**	**0.03**	**9.5**	**-0.04**	**0.01**	**5.0**	-0.9	0.7	1.2	-0.6	1.1	0.5
First fixation position squared (FP^2^)	**0.133**	**0.01**	**12.6**	**0.02**	**0.00**	**6.4**	**-4.9**	**0.3**	**18.2**	**-6.5**	**0.4**	**16.8**
G × FP	**0.098**	**0.04**	**2.7**	**-0.03**	**0.01**	**2.3**	-0.7	0.9	0.8	1.5	1.5	1.0
G × FP^2^	0.006	0.01	0.5	0.00	0.00	0.6	**3.6**	**0.3**	**11.9**	**3.5**	**0.5**	**6.6**
FP × FP^2^	-0.002	0.01	0.4	-0.00	0.00	0.1	**-0.5**	**0.1**	**3.9**	**-0.6**	**0.2**	**3.0**
G × FP × FP^2^	-0.001	0.01	0.2	-0.00	0.00	0.6	**-0.6**	**0.2**	**3.6**	0.5	0.3	1.7

*Reliable effects are shown in bold. FE: fixed effect; SE: standard error; z relates to Wald z which is a non-parametric statistic for the binomial or Poisson distributions of the re-fixation probability and the number of fixations, respectively.*

The right panels of **Figure 3** show the first fixation durations in relation to landing position. Note that, we distinguished between the first fixation durations of all cases and first fixation durations of cases in which the initial fixation was followed by at least one re-fixation indicating corrective re-fixations at unfavorable landing positions. In general, fixation durations of the slow readers were prolonged when compared to fluent readers. In relation to the landing position, the fixation durations of both groups were shorter after landing at the beginning or the end of a word in contrast to landing at the word center. Thus we observed an I-OVP effect for both measures and both groups. The main finding in **Figure 3** is that landing at the word end resulted in short fixation durations and, most critically, the durations were not different in the slow and the fast readers which was particularly the case for the fixation durations in multiple fixation cases.

The slow readers showed longer fixation durations than the fast readers for landing positions at the beginning and the center of the words. The LMM analysis (**Table 2**) confirms this observation. For both fixation duration measures, reliable effects of group and of the squared landing position were found. In addition, both first fixation duration measures showed a reliable interaction between the quadratic effect of landing position and group which was due to a more pronounced I-OVP effect in the slow than in the fast readers. The interaction between the linear and the quadratic landing position was also reliable indicating that an increase in the linear landing position effect was accompanied with an increase in the quadratic landing position effect. For first fixation duration only, a three-way interaction of reading group with the linear and the squared landing position was found. The interaction was due to fact that the slow readers exhibited both, a more pronounced linear reduction of fixation durations towards the word end and a more pronounced quadratic effect, than the fast readers.

**FIGURE 4:**
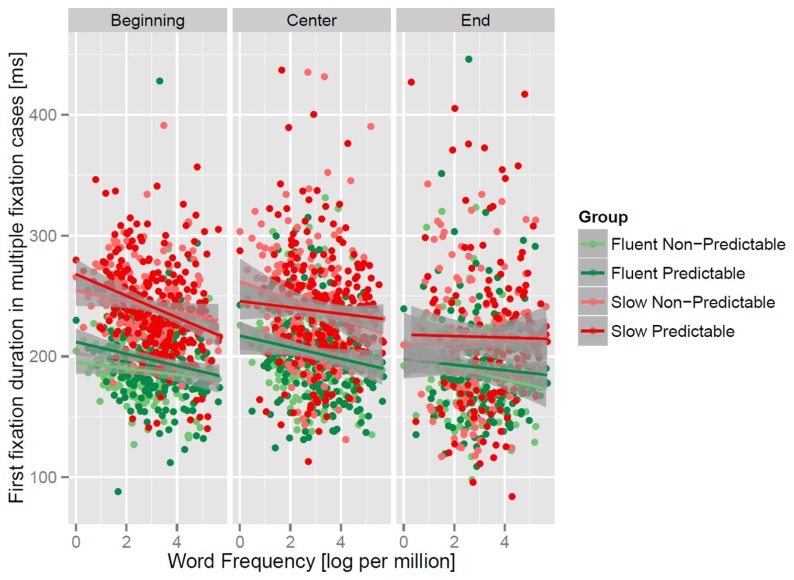
**The first fixation duration of multiple fixation cases in relation to word frequency for both groups, landing position and respective regression lines with confidence intervals for predictable (predictability > 0) and unpredictable words (predictability = 0).** The categorical distinction of predictability was only used for displaying the interaction of frequency and predictability; for the analysis the continuous predictability measure was used.

In an additional LMM analysis (**Table 3**; **Figure 4**), we investigated the influence of word frequency and predictability on fixation durations of multiple fixation cases separately for landing position (beginning, middle, end). This analysis is concerned with the question whether the short fixations after landing on the end of words are influenced by word frequency and predictability. The focus of this analysis was on the durations of the first fixation of multiple fixation cases. The rationale is that these fixations are the most sensitive measure to investigate corrective re-fixations. The analyses was a combined one for all levels of word length. A landing position of smaller than -2 was defined as landing at the word beginning and a landing position greater than +2 was defined as landing at word end. Landing positions between -2 and +2 were defined as landing at the word center. As evident from the regression lines in **Figure 4** (right panel) and the LMM analysis in **Table 3**, no reliable main effects or interactions of group, word frequency and word predictability were found when readers landed on the end of the words. This is a strong indication that linguistic processes did not influence fixation durations after landing on word ends. In contrast, if fixations landed at the beginning or the center of a word (left panel of **Figure 4**), then the slow readers exhibited longer fixation durations and we observed a reliable interaction of word frequency with group. This interaction was due to a more pronounced effect of word frequency in the slow compared to the fast readers. The predictability of the word influenced fixation durations only after landing on the word beginnings. A reliable predictability by frequency interaction which was due to a more pronounced effect of frequency in case of predictable words could be found at word beginnings.

**Table 3:** Results from LMM analysis for first fixation durations of multiple fixation cases with reading group, word frequency and predictability as fixed effects and participants and items as random effects.

	Landing position at word beginning			Landing position at word center			Landing position at word end		
	*FE*	*SE*	*t*	*FE*	*SE*	*t*	*FE*	*SE*	*t*
Group (G)	**-49.9**	**8.7**	**5.7**	**-69.1**	**7.4**	**9.3**	-14.6	13.5	1.1
Word predictability (P)	-42.1	23.2	1.8	-39.0	23.1	1.7	-62.1	52.0	1.2
Word frequency (F)	**-9.7**	**1.5**	**6.3**	**-11.0**	**1.5**	**7.6**	-2.2	3.3	0.7
G × P	54.4	31.7	1.7	35.5	29.5	1.2	25.2	58.3	0.4
G × F	**6.1**	**2.2**	**2.8**	**9.3**	**1.7**	**5.4**	-2.4	3.6	0.7
P × F	**14.3**	**6.2**	**2.3**	10.0	5.9	1.7	11.2	12.1	0.9
G × P × F	-11.4	8.7	1.3	-10.3	7.6	1.4	-3.0	13.6	0.2

*Reliable effects are shown in bold. FE: fixed effect; SE: standard error.*

### INDIVIDUAL EFFECTS

In addition to the group analysis, we conducted an individual estimation of the effect of squared landing position on fixation durations of multiple fixations, number of fixations and re-fixation probability. Separately, for each group, a simplified model was computed that included the centered linear and squared landing positions as fixed effects and participants and items as random effects on the intercept. In addition, the random effect of participants on the slope of the linear and squared landing position was included in the model. The additional random effects made possible to estimate the individual linear and quadratic effects of landing position. However, we will focus on the quadratic effects. The I-OVP effect on first fixation durations (i.e., a negative effect of the squared landing position) was found in 37 out of the 38 slow readers (97%) with a mean of -5.7 ms (SE = 1.9 ms; range: -10.1 to 1.6 ms). This effect was found in each of the fluent readers with a mean of -2.6 ms (SE = 1.0 ms; range: -4.9 to -0.1 ms). The OVP effect on number of fixations (i.e., a positive effect of the squared landing position) was found in 92% of slow (35/38) and 99% of fast readers (81/82) with means of 0.05 (SE = 0.5; range: -0.009 to 0.21 fixations) and 0.03 (SE = 0.2; range: -0.002 to 0.09 fixations), respectively. For the re-fixation probability, which showed the lowest quadratic effects of all three measures, only 71% (27/38) and 74% (61/82) of the slow and the fast readers showed an OVP effect. Mean values were 0.3% (SE = 1.3; range: -1.9 to 2.2 fixations) and 0.8% (SE = 1.3; range: -1.8 to 2.8 fixations), respectively.

A second analysis was conducted for each slow reader in respect to the first fixation durations of multiple fixation cases that landed on the word end. For these fixation durations the group analysis showed no reliable differences between the groups but this result might mask several slow readers that may still show increased fixation durations. In this analysis one sample t-tests were realized that compared the individual means of the slow readers compared with the fixation durations of the group of fluent readers (*M* = 201 ms; SD = 30). This analysis showed that 27 of the 38 slow readers exhibited first fixation durations which were not reliably different from the fluent readers. In other words, 71% of slow readers showed fixation durations (i.e., corrective re-fixations) comparable to those of the fluent readers.

## DISCUSSION

The present study investigated landing position effects in slow and fluent readers during sentence reading. We found (1) an OVP effect in re-fixation probability, (2) OVP in number of fixations and (3) an I-OVP effect on first fixation durations in slow readers. The main finding was that we found (4) no difference between slow and fluent readers in fixation durations at word end. Furthermore, the fixation durations of both groups were (5) not influenced by linguistic word characteristics, when the fixation landed at the end of a word. Thus, we can conclude that both groups exhibited a similar correction process (i.e., corrective re-fixations) after landing at unfavorable positions within words. However, the total number of fixations and the percentage of re-fixations were higher in slow readers compared to the fluent readers. Furthermore, we replicated the finding that slow readers initially fixate closer to word beginnings than fluent readers (MacKeben et al., 2004; Hawelka et al., 2010). A further group difference was that the I-OVP effect was stronger in the slow readers, which was reflected by prolonged fixation durations at the word center (and at word beginnings) accompanied by a steep decrease of fixation durations towards the word ends.

The I-OVP effect in fluent readers showed the expected pattern with the longest fixation durations at the center and shorter of fixation durations at suboptimal landing positions, that is, at the end and beginnings of words. In both groups the I-OVP effect was most pronounced in first fixation duration of multiple fixation cases and similar for all word lengths. Therefore, we speculate that in most of the multiple fixation cases at a suboptimal position the fixation duration reflect the visuo-oculomotor processes preceding a corrective re-fixation. These corrective re-fixations are the main objective of the study and hence we will focus on them forth on.

The group differences in fixation durations at the word center (i.e., the OVP) and word beginning (the preferred landing position in the slow readers) reflect linguistic processing. For these landing positions we found reliable effects of word frequency. The frequency effect was substantially more pronounced in the slower than in the fluent readers (replicating, e.g., Hawelka et al., 2010). After landing at the end of words, the fixation durations of both groups were similar and were not influenced by word frequency or word predictability. Therefore, fixation durations after landing at the end are not influenced by linguistic processes and the increased number of fixations and higher percentage for a re-fixation indicates that these fixations are highly likely followed by a corrective saccade towards the word center. When inspected in detail, these fixations were followed by a saccade towards the word center in 78 and 83% of the cases for fast and slow readers, respectively. Thus, one can assume that the pattern after landing at word end reflects corrective re-fixations, a mechanism which is intact in (the majority of) slow readers.

We observed group differences in fixation duration when the initial fixation was at the beginning of the words. The slow readers’ fixation durations were prolonged and more affected by word frequency than those of the fluent readers. This finding suggests that slow readers habitually target word beginnings (MacKeben et al., 2004). In fluent readers the preferred viewing location is slightly left to the word center (Rayner, 1979). However, even in the slow readers the fixation duration at word beginnings were, on average, shorter than fixations at the word center. Thus, the cohort of fixations at the beginnings of words might include two cohorts of fixations of different type: one small cohort in which the slow readers correct for suboptimal landing positions and a second, larger cohort which initialized the process of visual word recognition instantaneously (i.e., serial decoding; see below), that is, without correction of the landing position.

The differences in the fixation pattern might stem from differences in cognitive processes that lead to word recognition. In the study by Hawelka et al. (2010), the landing positions of dyslexic readers at the word beginning, in combination with their higher number of fixations and their strong word length effect, was interpreted as a reflection of word processing by means of serial decoding. In fluently reading adults, only words of very low frequency and pseudowords elicit a word length effect (Weekes, 1997). The present finding of the tendency of initially fixating at word beginnings accompanied with a second fixation at the word center, suggests that, at least in a considerable amount of cases, serial decoding is still present in our adolescent and adult slow readers.

The pattern of group differences suggests that slow readers show comparable corrective re-fixations than fluent readers, especially at word end; anyway, when linguistic processing is present, indicated by the frequency and predictability effects, their slow reading speed is reflected in massively prolonged fixation durations. On an individual level, the group pattern does not fit to all of the slow readers. The individual I-OVP effects on fixation duration and the OVP effect of number of fixations showed that a small number of slow readers (a maximum of 8%) did not show I-OVP and OVP effects. In comparison, all fluent readers showed an I-OVP effect and only one out of 82 fluent readers did not show an OVP effect on number of fixations. Only for the re-fixation probability OVP effect, which was the weakest of the three effects, a larger number of individuals were found that did not show a positive OVP effect. Here 26% of the fluent and 29% of the slow readers did not show an OVP effect. Although these percentages are high, they were comparable between the groups. Thus, the main finding from the individual analysis is that the vast majority of the slow readers do not exhibit visuo-oculomotor deficits and that deficient linguistic processing is the cause of their impaired reading speed. Studies, which assessed this issue with non-linguistic tasks, came to similar conclusions. Especially, sophisticated search tasks that used stimuli that were very similar to reading stimuli (e.g., consonant strings) found that slow and fluent readers showed comparable eye movement patterns (Hutzler et al., 2006; Prado et al., 2007) indicating that visual and oculomotor processing of the slow readers is intact. For the few slow readers who exhibited deviant I-OVP and OVP effects one could assume that non-linguistic processes be the proximal cause or an additional source for their slow reading speed. Although the prevalence of this type of deficit was very low in the present sample of slow readers, it deserves attention particularly with regard to individual diagnostic and individually tailored therapies.

To sum up, the present study on the I-OVP effect in first fixation durations and accompanying effects (e.g., OVP of number of fixations) informed about the influence of landing position on the eye movement characteristics of slow readers. In case of suboptimal landing positions both groups used a similar corrective mechanism, a fast corrective re-fixation to the word center. Similar corrective re-fixations in fluent and slow readers allow drawing the conclusion that visual and oculomotor processes cannot be the primary cause of the reading speed impairment.

## Conflict of Interest Statement

The authors declare that the research was conducted in the absence of any commercial or financial relationships that could be construed as a potential conflict of interest.
